# Alone on a collagen island: Unique findings of osseous sclerotic bodies in nephrogenic systemic fibrosis

**DOI:** 10.1016/j.jdcr.2023.08.010

**Published:** 2023-08-26

**Authors:** Emily R. Gordon, Megan H. Trager, Oluwaseyi Adeuyan, Celine M. Schreidah, Lauren M. Fahmy, Brigit A. Lapolla, Sameera Husain, Alexandra J. Coromilas, Larisa J. Geskin

**Affiliations:** a Columbia University Vagelos College of Physicians and Surgeons, New York, New York; b Department of Dermatology, Columbia University Irving Medical Center, New York, New York

**Keywords:** cutaneous findings, kidney disease, nephrogenic fibrosing dermopathy, nephrogenic systemic fibrosis, osseous sclerotic bodies, skin thickening

## Introduction

Nephrogenic systemic fibrosis (NSF) is a progressive disorder in patients with advanced kidney disease and is linked to the use of a specific isotope of gadolinium. Clinically, it presents with extremity and trunk skin thickening. Cutaneous findings in NSF consist of fibrotic, indurated papules, plaques, or subcutaneous nodules.^1^ Histologically, it is characterized by dermal expansion and fibrosis in association with CD34^+^ fibrocytes. Histopathology is critical to distinguish NSF from scleromyxedema, scleroderma, eosinophilic fasciitis, and spindle cell neoplasms. Osseous sclerotic bodies in NSF are rarely documented and clinical images and descriptions are lacking. We present a case of a 44-year-old woman with a history of longstanding NSF whose histopathology from biopsies, specifically from hyperpigmented plaques, revealed osseous sclerotic bodies.

## Case report

A 44-year-old woman with systemic lupus erythematosus presented with 2 years of “bruising” on her lower legs. Twenty-five years prior to this presentation, the patient developed lupus nephritis, which went into remission after treatment with cyclophosphamide and systemic steroids. Five years after her remission, she had an MRI with contrast during a hospitalization, which showed thickening and fibrosis of her skin. A skin biopsy was performed of her right forearm and hand, and results were consistent with nephrogenic fibrosing dermopathy or NSF.

For the past 18 years, the patient has reported that her legs have been “tight,” with skin thickening up to her knee but with no impact on ambulation or mobility. Two years prior to the current presentation, she developed “bruising” on the back of bilateral calves, and she was evaluated by hematology. It was suspected that the patient had cutaneous small vessel vasculitis secondary to systemic lupus erythematosus, and she was referred to dermatology.

**Fig 1:**
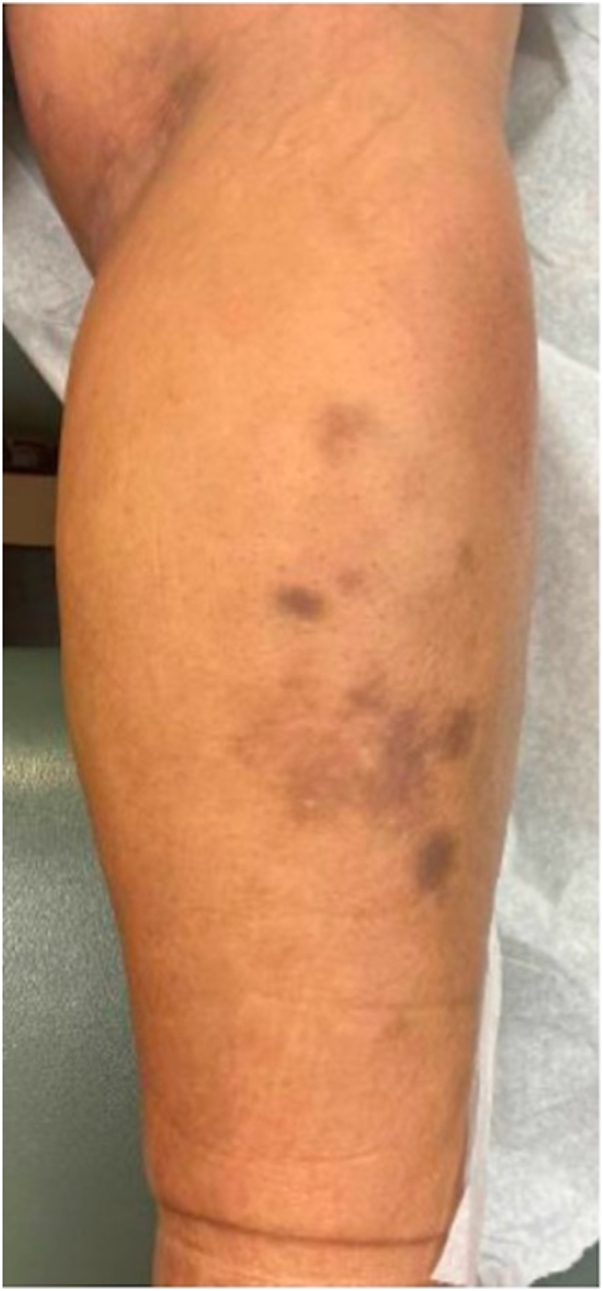
Clinical presentation, right posterior calf with purpuric plaques.

Upon evaluation by dermatology, physical exam was notable for lower leg sclerotic plaques and purpuric plaques on the posterior lower legs (Fig 1). Differential diagnosis was broad, including panniculitis (erythema induratum), vasculitis, postinflammatory hyperpigmentation from trauma or prior treatment with minocycline, or hemorrhage.

**Fig 2:**
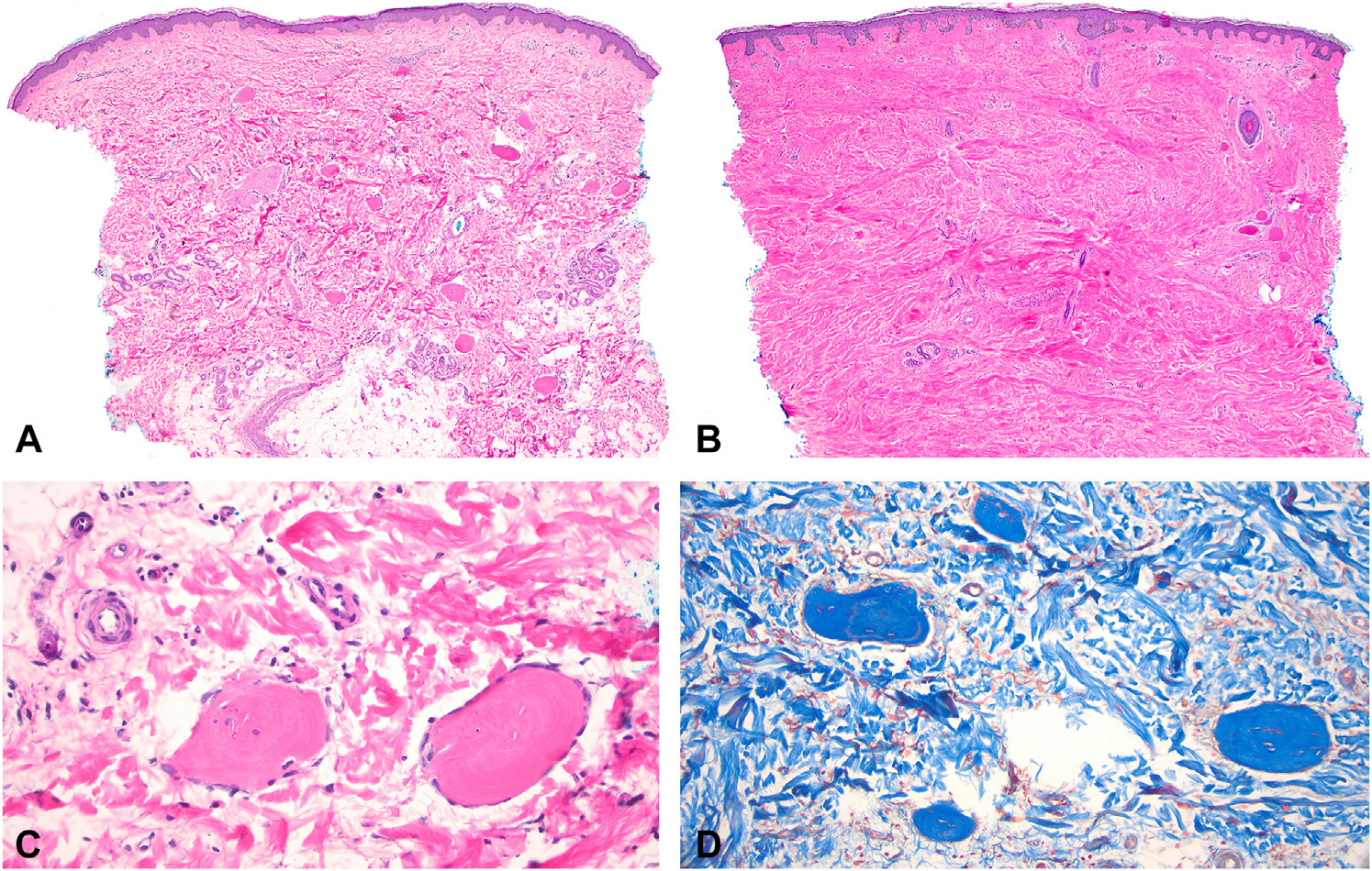
**A,** Hyperpigmented plaques, hematoxylin and eosin stain. A punch biopsy shows ovoid sclerotic bodies scattered throughout the dermis and within subcutaneous adipose tissue, with no significant sclerosis within the dermis (magnification × 40). **B,** Nonlesional skin, hematoxylin and eosin stain. A punch biopsy shows minimal sclerotic bodies on the periphery but significant sclerosis throughout the dermis (magnification × 40). **C,** Hyperpigmented plaques, hematoxylin and eosin stain. Osseous sclerotic bodies staining intensely *pink* (magnification × 400). **D,** Hyperpigmented plaques, trichrome stain. Osseous sclerotic bodies staining intensely *blue* (magnification × 200).

Skin punch biopsy of purpuric plaque on her legs showed ovoid sclerotic bodies (Fig 2, *C*) with occasionally trapped elastic fibers scattered throughout the dermis and within subcutaneous adipose tissue, with no significant sclerosis within the dermis (Fig 2, *A*). Osseous sclerotic bodies stained intensely blue with a trichrome stain, highlighting their sclerotic nature (Fig 2, *D*). A biopsy of nonlesional fibrotic skin, compared to the previous biopsy, demonstrated minimal osseous sclerotic bodies at the lateral margin of the biopsy but a significant amount of sclerosis throughout the dermis, consistent with NSF (Fig 2, *B*).

About 1 month later, the patient developed asymptomatic hyperpigmented patches on her left arm, and a skin biopsy was performed. Biopsy showed mild chronic inflammation, dermal fibrosis with spindle cell prominence, and rare osseous sclerotic bodies in the deep dermis, consistent with NSF. The patient underwent workup including iron panel, metabolic panel, urinalysis, complement levels, blood count, and antibody assessment for autoimmune disease such as systemic sclerosis including anti-centromere, anti-topoisomerase 1, and anti-RNA polymerase III, all of which were unremarkable. The patient was treated with topical tacrolimus 0.1% ointment and intralesional injections of triamcinolone 5 mg/mL (total volume 0.3 mL) and experienced clinical improvement.

## Discussion

We present an unusual clinical presentation of NSF with osseous bodies seen on histopathology. Typical skin biopsy findings of NSF include increased fibrocytes and mucin deposition and thickening of collagen bundles.^2^ Osseous sclerotic bodies in NSF are a rare finding, with minimal clinical and histopathological images and descriptions in the literature. The term “sclerotic bodies” was originally used by Bhawan et al to describe islands of amorphous hyalinized collagen, mixed with trapped elastin fibers. They detailed 2 cases of NSF with these findings and distinguished them from osseous metaplasia, which has lacunae and cells within the osseous bodies that may or may not be calcified. Clinically, one patient had woody induration of the lower legs and fibrosis and brawny hyperpigmentation and flexion contractures of the fingers. The second patient had hyperpigmentation over her legs and forearms with peau d’orange changes.^3^ A subsequent report by Bhawan et al^4^ included a case with an unremarkable clinical picture, without hyperpigmentation, induration, or contractures, but with oval sclerotic bodies with central ossification on pathology. Each of these reports supports the conclusion that osseous sclerotic bodies may be an additional clue in diagnosing NSF.

Cutaneous symptoms vary between early and late stage disease in NSF. Description of late stage cutaneous findings in NSF are limited. The skin manifestations in NSF range from early stage of the disease with erythema, edema, and warmth to the late stage of disease as in this patient who was seen 18 years after initial symptoms began. Bangsgaard et al^5^ examined late skin manifestations of NSF in 17 patients and found a wide variety of clinical presentations. As in the current case, all patients had lower leg involvement. Superficial skin changes included epidermal atrophy, hair loss, hyperpigmentation, hyperkeratosis, and peau d’orange changes of the skin. Deep skin changes were varied, though a majority of the patients presented with areas of confluent dermal plaques of thickening and hardening. A few patients, however, had a cutis laxa type phenotype with wrinkled and redundant skin. In our case, the hyperpigmented purpuric plaques were likely a cutaneous symptom of late stage NSF given her history of skin thickening for the past 18 years.

The cause of osseous sclerotic bodies is unknown but may indicate progression of skin lesions over time rather than recurrence or recrudescence of NSF. In terms of etiology, her autoimmune workup and assessment of activity of systemic lupus erythematosus was unremarkable, suggesting that recurrence of lupus nephritis was not a trigger of recrudescence of skin lesions. Thus, the patient’s skin findings, such as hyperpigmentation on the legs, were most likely a late clinical manifestation of her NSF. Osseus sclerotic bodies have been shown to contain the nonradioactive heavy metal gadolinium, which has a known association with NSF.^6^

Overall, we present rare findings of osseous sclerotic bodies in NSF. The unique clinical scenario, images, and histopathology contribute to the literature on the etiology of this condition, diagnosis, and treatment.

## Conflicts of interest

Dr Geskin has served as an investigator for and/or received research support from Helsinn Group, J&J, Mallinckrodt, Kyowa Kirin, Soligenix, Innate, Merck, BMS, and Stratpharma; on the speakers’ bureau for Helsinn Group and J&J; and on the scientific advisory board for Helsinn Group, J&J, Mallinckrodt, Sanofi, Regeneron, and Kyowa Kirin. Author Gordon, Dr Trager, Author Adeuyan, Author Schreidah, Author Fahmy, Author Lapolla, Dr Husain, and Dr Coromilas have no conflicts of interest to declare.
